# Advances in the Physico-Chemical, Antimicrobial and Angiogenic Properties of Graphene-Oxide/Cellulose Nanocomposites for Wound Healing

**DOI:** 10.3390/pharmaceutics15020338

**Published:** 2023-01-19

**Authors:** Ugo D’Amora, Sawsan Dacrory, Mohamed Sayed Hasanin, Angela Longo, Alessandra Soriente, Samir Kamel, Maria Grazia Raucci, Luigi Ambrosio, Stefania Scialla

**Affiliations:** 1 Institute of Polymers, Composites and Biomaterials, National Research Council (IPCB-CNR), 80125 Naples, Italy; 2 Cellulose and Paper Department, National Research Centre, 33 El Bohouth St., Cairo 12622, Egypt

**Keywords:** cellulose, graphene oxide, antimicrobial, angiogenesis, wound healing

## Abstract

Graphene oxide (GO) and its reduced form (rGO) have recently attracted a fascinating interest due to their physico-chemical properties, which have opened up new and interesting opportunities in a wide range of biomedical applications, such as wound healing. It is worth noting that GO and rGO may offer a convenient access to its ready dispersion within various polymeric matrices (such as cellulose and its derivative forms), owing to their large surface area, based on a carbon skeleton with many functional groups (i.e., hydroxyl, carboxyl, epoxy bridge, and carbonyl moieties). This results in new synergic properties due to the presence of both components (GO or rGO and polymers), acting at different length-scales. Furthermore, they have shown efficient antimicrobial and angiogenic properties, mostly related to the intracellular formation of reactive oxygen species (ROS), which are advantageous in wound care management. For this reason, GO or rGO integration in cellulose-based matrixes have allowed for designing highly advanced multifunctional hybrid nanocomposites with tailored properties. The current review aims to discuss a potential relationship between structural and physico-chemical properties (i.e., size, edge density, surface chemistry, hydrophilicity) of the nanocomposites with antimicrobials and angiogenic mechanisms that synergically influence the wound healing phenomenon, by paying particular attention to recent findings of GO or rGO/cellulose nanocomposites. Accordingly, after providing a general overview of cellulose and its derivatives, the production methods used for GO and rGO synthesis, the mechanisms that guide antimicrobial and angiogenic processes of tissue repair, as well as the most recent and remarkable outcomes on GO/cellulose scaffolds in wound healing applications, will be presented.

## 1. Introduction

Surgery, trauma, infection, diabetes and other diseases are among the most common causes of a wound genesis. In particular, skin wound healing is a complex phenomenon upregulated by different cell types and based on a fine orchestration of biological and molecular events, such as cell migration, proliferation, and extracellular matrix (ECM) remodelling [1]. In chronic wounds, sustained inflammation, continuous infections, antibiotic-resistant biofilms development, as well as the incapability of resident cells to reply to reparative stimuli may compromise the overall process.

In this scenario, over the past years, chronic wounds have become a life-threatening issue. Nowadays, worldwide, approximately 10 million people experience chronic wound (i.e., burning), leading to death if not properly handled [2]. For this reason, in the management of those injuries, selecting a suitable wound dressing is crucial. By this point of view, material choice is of paramount importance to ensure an appropriate mechanical stability to support skin injuries, a suitable water retention to absorb exudates, promising antibacterial properties to prevent biofilm formation, and tear resistance. Importantly, the material should be able to promote the healing by guaranteeing a long-term drug and growth factors (GFs) delivery with a controlled biodegradation, as well triggering the angiogenesis [3]. Ointments in the form of cream or natural oils, gels, gauzes, and bandages are among the wound dressings currently available, due to their unique combination of high water content, softness, flexibility, and biocompatibility [4,5,6,7,8]. They generally consist of natural (e.g., chitosan, cellulose, keratin, casein, collagen, hyaluronic acid, alginate, and silk fibroin) and/or synthetic (e.g., polyvinyl alcohol, polyethylene glycol, poly[lactic-co-glycolic acid], polycaprolactone, polylactic acid) polymers, and also bioactive compounds (e.g., chemical drugs, metallic nanoparticles, GFs, stem cells, and plant extracts) [9]. Furthermore, the advent of smart wound dressings by using 3D printing has revolutionized wound care management [5,10,11].

Among the natural polymers, cellulose is emerging as a suitable candidate for wound healing applications, where flexibility and toughness are of paramount importance together with biocompatibility and antibacterial properties. Furthermore, it has excellent properties, including tunable mechanical strength, high purity, good water retention, high specific surface area, biodegradability, and low toxicity [12,13,14]. The three hydroxyl (-OH) groups in the monomer units, which repeat along the cellulose backbone, are involved in the formation of hydrogen bonds, directing the crystalline regions and controlling the physico-chemical properties. These -OH groups also provide a high reactivity to cellulose towards chemical modification processes, such as carboxymethylation, acetylation, oxidation and silylation [15], to obtain cellulose derivatives useful for medical applications and metals or other inorganic precursors absorption. 

However, cellulose naturally lacks antimicrobial properties. Among the antibacterial agents, graphene oxide (GO) and its reduced form (rGO) have recently gained enormous interest in biomedical applications. Studies on antibacterial properties of GO-like materials are contradictory and have raised confusion. Several groups of researchers reported GO and rGO to possess bactericidal properties against both Gram-positive (Gram^+^) and Gram-negative (Gram^−^) bacteria. Some works suggested that GO has no effect on bacteria, or in opposition, other papers highlighted GO and rGO as enhancers of microbial growth for *Escherichia coli*. However, it is stated that cell growth has been prevented for Gram^+^ bacteria, whereas, on Gram^−^ bacteria, GO-like materials have no effect [16]. These inconsistent results have led to the scope of the present detailed overview, whether GO or rGO-based cellulose biomaterials for wound healing applications, have been analysed in order to find a possible explanation of their action in the destruction of bacteria cells. Indeed, the presence of the polymer matrix can dramatically alter the possible antibacterial potential of GO or rGO. At the point of the present state of art, it is rather complex to give a comprehensive review of all the literature concerning GO or rGO/cellulose nanocomposites for wound healing applications.

In this review, the aim of the present work was to critically discuss the potential relationship between structural and physico-chemical properties of GO or rGO/cellulose nanocomposites on the antimicrobials and angiogenic mechanisms, which synergically regulate the wound healing. This review will then give an overview of: (i) the cellulose-derivatives and related production processes, with a special focus on the agricultural wastes, in view of a more circular and green economy, (ii) GO and rGO synthesis, and (iii) the antimicrobial and angiogenic pathways involved in the wound repair. (iv) To finish, the most recent and remarkable findings of the last 3 years on the multifunctional GO or rGO/cellulose biomaterials, in the form of scaffolds and injectable hydrogel formulations, intended for wound healing, will be presented.

## 2. Cellulose and Cellulose Derivatives

**Figure 1:**
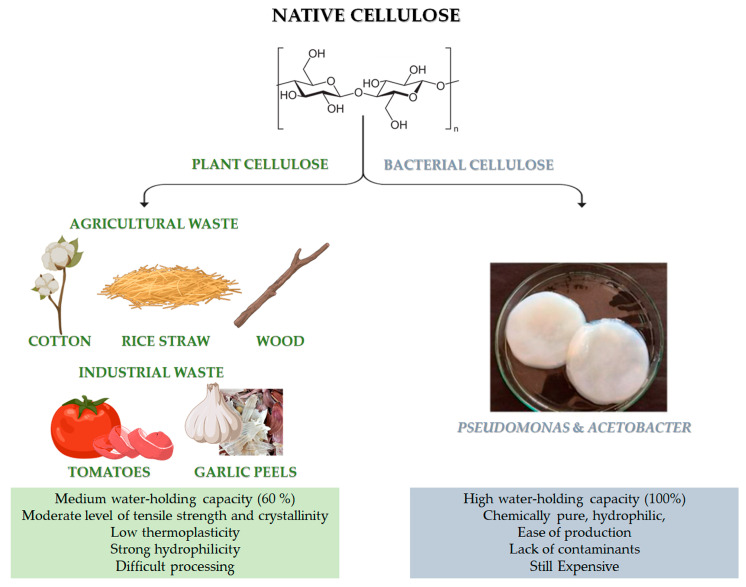
Chemical structure of cellulose and different sources of: native cellulose (from agricultural and industrial wastes) and bacterial cellulose (BC). Advantages and disadvantages of both kinds of cellulose. The image of BC is adapted from [18]. Copyright 2022 by the authors. License MDPI, Basel, Switzerland.

Among the biomaterials, cellulose is the most abundant, cheap, sustainable, chemical reactive and modifiable natural macromolecular compound on the Earth. It is a carbohydrate homopolymer, which is composed of repeating long linear chains of *β*-anhydro-D-glucopyranose units, linked together by an ether bond between -OH group of C4, and the C1 carbon atom, via a *β*-1,4-glycosidic bond, as represented in Figure 1 [17].

Native cellulose can be mainly classified into plant-derived and bacterial-derived cellulose (BC) [19]. It is worth noting that cellulose can be also obtained by animal sources [20]. In particular, some marine animals, called tunicate, represent the only animal source of cellulose [20]. However, it is still limited and not available for large-scale applications. Naturally-derived cellulose is a versatile, structural polysaccharide polymer, which mainly provides robust mechanical properties to plant cells, thanks to the hierarchical organisation of its natural fibres [21]. However, the structural backbone skeleton of plants represents a non-pure formulation that is usually based on lignocellulose, consisting of lignin, cellulose, hemicellulose, silica and some other impurities with different ratios, according to the plant type, as reported in Table 1. Indeed, plant cellulose widely exists in cotton, wood, and other flora species, such as phloem fibre, seed fibre, and wood fibre, which is the most abundant organic substance in nature [22]. Native cellulose also has the benefit of being able to be derived from agricultural wastes, such as cotton, bamboo, bagasse, and rice straw, which are affordable, readily available, and sustainable material sources [23]. Moreover, food processing is another possible source for cellulose recovery, even though it has highlighted many restrictions related to the solid waste collectability and the status of these wastes after gathering, which are usually contaminated with microorganisms and other impurities. Lately, industrial wastes (i.e., tomato, garlic peels) are also emerging as a promising source of cellulose.

Recently, bacterial cellulose has gained particular interest [24]. BC can be produced by strictly aerobic, non-photosynthetic Gram^−^ bacteria [24,25]. For their growth, these bacteria need carbon sources, which can be found in different raw materials (such as fruit, vegetable or lignocellulosic wastes) and other nutrients (such as nitrogen, iron, zinc and vitamins) [24,26]. *Pseudomonas*, *Gluconacetobacter* and *Acetobacter* are among the most important bacteria for the BC synthesis (Figure 1). Numerous studies have been carried out on the potential benefits of BC and plant-derived cellulose as biomaterials. In particular, the macromolecular characteristics of bacterial- and plant-derived cellulose differ. In addition, 60% of plant-derived cellulose can hold a medium amount of water, and it has a medium level of tensile strength and crystallinity. Conversely, BC is chemically pure, due to its hydrophilic nature, 100% water-holding capacity and lack of lignin, hemicellulose, and other impurities [22]. Furthermore, compared to plant-derived cellulose, BC has high crystallinity [22]. However, BC is still a high cost cellulose if compared to other conventional cellulose [27]. The versatility of BC’s biomedical uses is supported by its simple, contaminant-free manufacture and flexibility to modify the material’s properties during synthesis, such as crystallinity index, aspect ratio, and morphology, to precisely meet the needs of the intended application [22].

**Figure 2:**
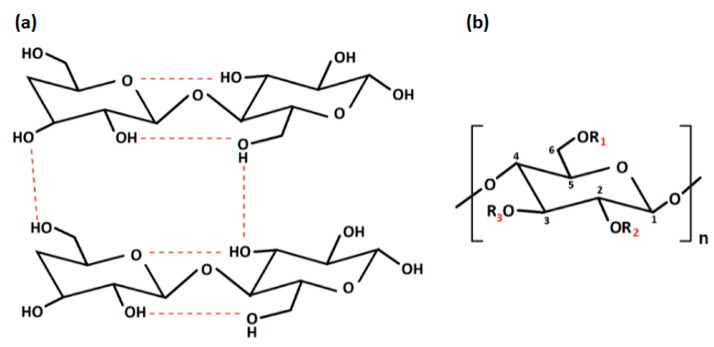
(**a**) Intra- and inter-molecular hydrogen bonds in cellulose; (**b**) repeating unit of cellulose derivatives. “Created with BioRender.com“.

Native cellulose has multiple shortcomings, such as poor solubility in water and most organic solvents, due to intra- and intermolecular hydrogen bonds, low thermoplasticity, strong hydrophilicity, and difficulty in processing, which limit its development and application in the biomedical and pharmaceutical fields [22]. The intra- and inter-molecular hydrogen bonds, between the -OH groups of the chain, provide cellulose with a crystalline and stiff structure. For this reason, it is insoluble in water but can be dissolved in strong acidic or alkaline conditions [28] (Figure 2). In particular, cellulose cannot dissolve in common solutions, except for two kinds of solvents: non-derivatizing solvents (i.e., sodium hydroxide, melts of inorganic salts, hydrates of inorganic salts, N, N-dimethylacetamide/lithium chlorideandmineral acids) and derivatizing solvents (i.e., trifluoroacetic acid, formic acid, dimethyl sulfoxide/para-formaldehyde, N, N-dimethyl formamide/dinitrogen tetroxide) [29]. Fortunately, insolubility can be overcome by obtaining cellulose derivatives, also known as cellulosics, through various physical and chemical modification procedures, such as esterification, etherification, or oxidation [30]. By this point of view, cellulose is the most known polysaccharide that can be easily converted to many cellulose derivatives. Among them, there are cellulose derivatives, which can be non-soluble, soluble in organic solvents and water-soluble, with a high safety profile towards biological systems [31].

### 2.1. Cellulose Physical Modifications

By altering the structure and surface properties of cellulose, physical modification is mostly used to create new qualities and functions. Briefly, the physical alteration largely entails mechanical swelling, recombining, surface adsorption, and grinding without changing the chemical composition of cellulose. Physically-modified cellulose forms are regenerated cellulose, membrane cellulose, microcrystalline cellulose (MCC), spherical cellulose, and nanocellulose (NC) in its two categories: cellulose nanocrystals (CNC) and cellulose nanofibers (CNF) [21]. NC is attracting a wide research attention for its physico-chemical properties: high specific surface area, easy modification, biodegradability, non-toxicity, biocompatibility, and antimicrobial properties, which are making it a suitable biomaterial for biomedical applications (i.e., wound healing).

### 2.2. Cellulose Chemical Modifications

With regard to the chemical modification, two different types of reactions are involved: -OH groups derivatisation and cellulose degradation. Acid/base, oxidative, biological, and mechanical processing are all examples of degradation reactions [22]. On the other hand, derivatisation may provide the synthesis of useful chemical products. At a macromolecular level, cellulose is characterised by both crystalline and amorphous regions. The crystalline region is distinguished by densely and firmly packed -OH groups that become unavailable. For this reason, this region is less reactive in comparison with the other one. The amorphous region is more available with high reactivity toward chemical species. Indeed, the majority of the chemical modifications involve the amorphous region [32]. Moreover, cellulose chains are linear, and their aggregation occurs via both intra- and inter-molecular hydrogen bonds as presented in Figure 2a, affecting its degree of crystallinity. This aspect is of paramount importance as the physico-chemical properties of cellulose are fine-tuned by the degree of crystallinity, as well as the hydrogen bonding [33]. Additionally, the reaction behaviour of cellulose and accessibility depend on cellulose morphology, degree of polymerization, crystallinity degree, purity and particle size. It is worth noting that cellulose units have three active -OH groups C6 > C2 > C3, with the C6–OH group, which can react ten times faster than the other two groups C2 and C3 (Figure 2b).

Modification via carboxymethylation, oxidation and Micheal addition reaction can introduce new functional groups in the cellulose backbone including -OH, carboxyl (-COOH), cyano (-C=N), aldehyde (-CH=O) and tetrazole groups, leading to derivatives, such as carboxymethyl cellulose (CMC), methylcellulose (MC), dialdehyde cellulose (DAC) and hydroxyethyl cellulose (HEC), as summarised in Table 2. Over the past years, because of the increasing demand for environmentally eco-friendly and biocompatible products, the possibility to chemically modify the cellulose has boosted great advances in material science and engineering, leading to the use of cellulose derivatives in promising fields of applications, such as pharmaceutical and biomedical [34], but also electronic [35,36], as well as a water treatment one [37,38]. 

Cellulose derivatives are semi-synthetic highly water-soluble biopolymers, having high biocompatibility, biodegradability, non-toxicity, non-immunogenicity properties [39], and also a thermo-gelling behaviour [40]. In addition, cellulose derivatives can absorb and retain a large amount of wound exudates within the interstitial sites of the matrixes, maintaining an optimal local moisture at the lesion site, to avoid skin tissue water loss and tissue necrosis [39,40]. Therefore, they represent a good alternative to water-insoluble cellulose. However, mechanical strength and toughness continue to be the most difficult problems, which scientists have attempted to solve in a variety of ways: (i) by the use of non-covalent (i.e., citric acid, polyphenols) or covalent (i.e., epichlorohydrin, aldehyde-based reagents, urea derivatives, carbodiimides and multifunctional carboxylic acids) crosslinking agents [41], (ii) by designing blends with other synthetic (i.e., polyurethane, poly (vinyl alcohol), polyvinylpyrrolidone) [42] or natural (i.e., collagen, gelatin, chitosan, k-carrageenan, alginate) biopolymers [43]; (iii) by employing gelling agents [44]; (iv) by using nanomaterials as fillers (i.e., graphene oxide derivatives, titanium oxide, silver nanoparticles, zinc oxide, ceramics) [45,46,47].

### 2.3. Cellulose from Agricultural Waste: Extraction Methods and Properties

Nowadays, taking into consideration the environmental problems arising from a rapidly growing industry, the demand of products obtained starting from renewable and sustainable non-petroleum-based resources is increasing. For this reason, exploring naturally-derived biomaterials, such native cellulose extracted from agricultural wastes, is attracting outstanding attention in the scientific community. Extraction of cellulose can be carried out using different approaches that are mainly based on chemical, biological and physical methodologies [48]. In this context, the chemical method is considered as the best approach, in terms of productivity and low-time consumption. However, low sustainability is among the main drawbacks [49,50]. Additionally, the biological method deals with microbial enzymes, such as ligninolytic enzymes, lignin peroxidases and laccases enzymes, which are able to extract cellulose [51,52]. This approach is highly eco-friendly but has many limitations, such as having low productivity, specificity, high-cost facilities and being time-consuming. The physical method specifically employs a mechanical treatment for lignocellulosic materials to produce high lignin content pulp that is used in specific applications not as pure cellulose [53]. However, each extraction method can build up a unique cellulose in terms of properties and reactivity. The chemical method of cellulose extraction usually produces cellulose contaminated with trace elements that can be found in a cellulose molecular structure during the pulping and the bleaching process, such as sulphur, chlorine, sodium and iodine. On the other hand, this method produces cellulose with good chemical properties with different shapes, including MCC, cellulose fibres and cellulose nanoforms [54]. The biological method overcomes the drawback of the chemical method, obtaining highly pure products. Finally, the physically extracted cellulose enables the production of a material with a high lignin content pulp, which is mainly used in packaging and high mechanical stress applications [55].

## 3. The Graphene Oxide and Reduced Graphene Oxide

Graphene oxide (GO) is an exceptional 2D material consisting of a single layer of carbon atoms with oxygen-containing functional groups (=O, -OH, -O-, -COOH). Nowadays, it represents a great precursor for preparing reduced-graphene oxide (rGO) [56,57]. Therefore, GO, obtained from the almost complete removal of the oxygen functional groups [56], has mechanical, electrical, and optical properties quite similar to graphene, thus resulting in a convenient 2D material useful for many technological applications [58,59,60,61,62,63]. Due to its hydrophilic nature, GO can be stably and homogeneously dispersed in aqueous or polar organic solvents, in the form of colloidal suspensions, allowing an easy cast process of new devices, such as transparent conductive films and sensors [64,65,66]. With a high lateral dimension-to-thickness ratio, an amphiphilic nature due to its many surface functional groups, chemical inertness, and remarkable mechanical qualities, GO combines a number of beneficial properties [64]. In addition, GO is characterised by a large surface area, high thermal conductivity, high drug loading efficiency, bioactivity [47], biocompatibility, and biodegradability [67,68]. Therefore, it has been studied for tissue regeneration [69], gene therapy [70], photothermal treatment [71], and biotechnology techniques such as proteomics [72] and adsorption [73], as an antimicrobial platform [71]. Unfortunately, it is difficult to explain the precise chemical structure of GO and therefore of rGO, due to the irregular density of the carbon-skeleton defects and the different oxygen-containing groups present on the skeleton. The most acceptable structural model proposed for GO is due to Lerf-Klinowski [74], in which the basal planes of carbon atoms are decorated by -OH and epoxide (R−O−R’) groups, whereas the edges are mainly occupied by -COOH and carbonyl (–C=O) groups in a random manner [75,76,77,78]. By contrast, rGO can be described as a random distribution of residual oxidized regions, combined with non-oxidized ones [75]. It is well known that the reduction of GO has the effect not only of removing the oxygen-containing groups, but also on repairing some defects, while restoring the conjugated graphitic network [75]. Furthermore, due to the structure deformation and the presence of covalently bonded oxygen-containing groups, the GO layer is atomically rougher than the rGO layer, which appears as a more rigid structure in the basal plane [64,79]. However, GO and rGO sheets show a graphene-like honeycomb lattice, characterised by disorder and deformation [80]. The presence of the oxygen functionality affects the wettability of the GO, and hence the interaction with biological moieties. Several recent theoretical studies demonstrated that highly functionalized GO interacted most favourably with the biomolecules via hydrogen bonding, whereas the GO with low oxygen functional group densities prevalently interacted through π−π stacking [81,82,83,84,85,86,87]. Furthermore, the oxidation and reduction grade of GO sheet are strictly related to the oxidation and reduction method, respectively, whilst the number and the type of the oxygen-containing groups are strictly connected to the graphite precursor. Therefore, GO and rGO properties can be tailored by varying the graphite precursor with different size and number of defects.

### 3.1. Conventional Synthesis of Graphene Oxide

GO is produced from graphite by means of a two-step approach. Firstly, the graphite crystals are oxidized with strong oxidizing agents (i.e., H_2_SO_4_, KMnO_4_), introducing oxygen-containing functional groups. Secondly, the obtained “graphite oxide” adopts oxygen-containing groups, which facilitate the water-dispersion through sonication, thus increasing interlayer distance. Finally, graphite oxide can be exfoliated into either single or multilayers of oxygen-functionalized GO. Brodie was one of the first scientists who described a method to produce GO, where fuming HNO_3_ and KClO_3_ were used as intercalation and oxidative agent of natural graphite, respectively [88]. Later, the Staudenmaier method improved Brodie’s one by creating GO with a better C/O ratio by usingH_2_SO_4_ and HNO_3_ solutions [89]. These two techniques, however, rely on a drawn-out oxidation process that can take up to a week. One more method to produce GO was developed by Hummer, in which H_2_SO_4_ and NaNO_3_/KMnO_4_ were used by completing the oxidation reaction within 2 h [90]. In the last few years, many studies have been focused on improving the methods for GO synthesis by using eco-friendly conditions in order to maximise GO yields.

### 3.2. Synthesis of Graphene Oxide by Waste

**Figure 3:**
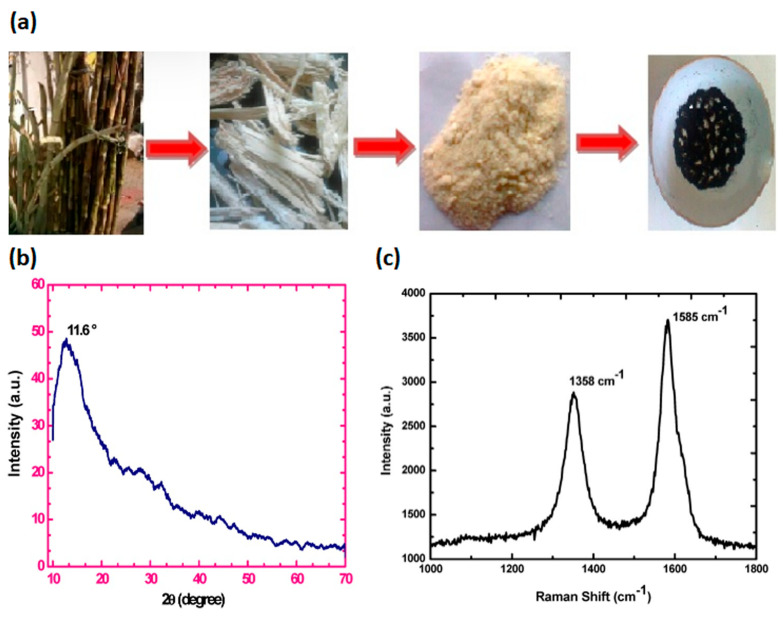
(**a**) Graphic scheme of sugarcane bagasse wastes conversion to GO; (**b**) X-ray diffraction (XRD) pattern; and (**c**) Raman spectrum of bagasse waste-derived GO, adapted from [91]. Copyright © 2015 by the authors; licensee MDPI, Basel, Switzerland.

In view of the last sustainable development trends, based on the new paradigm “reduce, re-use, and retain”, the valorisation of wastes may constitute an interesting platform in the circular bio-economy for the development of high added-value compounds, such as bioactive compounds and nanomaterials, with remarkable financial and environmental advantages, especially dealing with the problems associated with local and global pollutions. In this section, a summary of the most recent literature advances, focused on graphite extraction from agricultural or industrial waste-biomass, is reported and listed in Table 3. Different natural wastes, such as wood, leaf, sugarcane bagasse [91], tea waste biomass [92] and coconut shell [93], have been employed by a carbonization process for charcoal precursors preparation, to synthesize GO. Somanathan et al. produced GO via single-step reforming of sugarcane bagasse agricultural waste by oxidation under muffled atmosphere conditions [91]. In particular, sugarcane bagasse fine powder was mixed with ferrocene and thermal treated into a muffle furnace at 300 °C for 10 min under atmospheric conditions, outlined in Figure 3a. The successful conversion of solid sugarcane bagasse waste into GO nanosheets was confirmed by morphological and structural investigations (Figure 3b,c).

**Figure 4:**
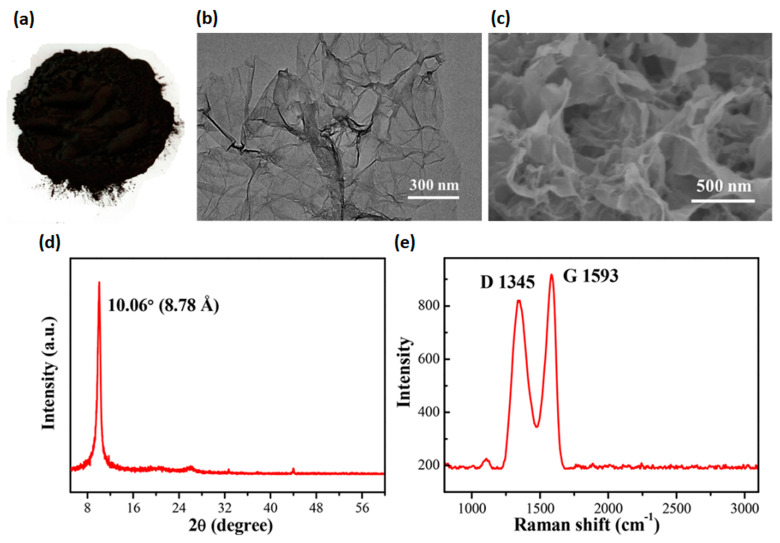
(**a**) Optical photograph; (**b**,**c**) SEM images of 3D porous GO with different magnifications; (right) TEM images with different magnifications; (**d**) XRD and (**e**) Raman spectrum. Reprinted with permission from [95]. Copyright 2022 American Chemical Society.

Recently, Faiz et al. produced GO by conversion of tea waste biomass via carbonization process at high temperature [92]. The following oxidation and exfoliation of graphitized carbon was performed by using an adapted Hummer’s method [90]. The oxidation of the graphitized tea waste was confirmed by the presence of oxygen-containing functional groups (i.e., OH, C=O and C–O). Meanwhile, coconut shell wastes were also employed by Sujiono and co-workers to synthesize GO through a modified Hammer’s method [90]. Physico-chemical analysis confirmed the GO formation due to the presence of various oxygen-containing functional groups within the structure, while the XRD pattern showed that 71.53% of graphite 2H observed with GO sample tended to form a rGO phase [93]. Lately, industrial wastes have gained growing interest as alternative raw materials for carbonaceous precursors production, through the removal of hazardous metal impurities. The possibility to re-use carbon tyre waste as a precursor to synthesize the GO by Hummer’s method was analysed by Bonnaia et al. [94]. Raman spectra characterized by two peaks that can be referred to as D band and G band, located at 1361 cm^−1^ and 1596 cm^−1^ with the intensity ratio of the D band relative to the G band (I_D_/I_G_) being 0.88, confirmed the obtained GO. Similarly, Tian et al. used toner powder waste as a precursor of GO (Figure 4a) through a one-pot adapted Hummer’s method [95]. A 3D porous GO with excellent morphology and microstructure was obtained. In particular, several GO sheets well-assembled and interconnected to form a “cotton-like” 3D structure with wrinkling and “waviness” were observed through scanning electron microscopy (SEM) and transmission electron microscopy (TEM) (Figure 4b,c). The XRD pattern exhibited the characteristic diffraction peak of GO at 2θ = 10.06° (Figure 4d), which was consistent with a lamellar distance of ~8.78 Å. The interlayer spacing of the GO was larger than graphite (3.35 Å), arising from an intercalation of oxygen-containing groups between the GO sheets. Meanwhile, the characteristic D peak (~1345 cm^−1^) and G peak (~1593 cm^−1^) of GO were further observed via Raman analysis (Figure 4e). Siaw et al. produced the GO, with a purity of 92.28%, through a leaching process to remove most of the impurities by industrial waste. Subsequently, GO was synthesised via a modified Hummers method with a combination of concentrated H_2_SO_4_ and KMnO_4_ [96]. 

## 4. Short Overview of GO-Derivatives’ Role in Antimicrobial Activities and Angiogenic Mechanisms 

### 4.1. Antimicrobial Mechanisms Involving GO-Derivatives

The pristine graphene, GO and rGO differently interact with bacteria because of their structural and physico-chemical differences. Graphene-bacteria membrane interaction is strongly influenced by surface charge, which is neutral in the pristine graphene, while negatively charged in GO and rGO. However, several aspects need to be considered when assessing the graphene’s antimicrobial action: the physico-chemical properties (i.e., size, number of layers, shape, surface modifications, agglomeration and dispersion), as well as the microorganism category (Gram^+^/Gram^−^), shape (rod/spherical), type (aerobic/anaerobic) and stage of maturity. Moreover, differences in graphene inherent properties may arise from the synthesis processes or the (un-)controlled functionalization mechanisms, leading to disputable cytotoxic findings [97]. Similarly, the bacterial cell envelope’s complexity also plays a decisive role. Gram^+^ bacteria own a thicker (20–80 nm) layer of peptidoglycan, surrounding the phospholipid bilayer and acting as barrier towards osmotic pressure changes and harmful molecules; while Gram^−^ bacteria hold a thinner (2–8 nm) layer of peptidoglycan [98,99]. Therefore, *Staphilococcus aureus* (Gram^+^ bacteria) is more susceptible to graphene than *Pseudomonas aeruginaosa* (Gram^−^ bacteria) [100,101]. In addition, Gram^+^ bacteria interact with graphene through hydrogen bonding, *π*-*π* stacking and electrostatic adsorption with the main components of the peptidoglycan [102,103], causing morphological deformation in the membrane [100,101]. Conversely, graphene interacts with Gram^−^ bacteria via direct contact, leading to membrane disruption [100,101]. 

**Figure 5:**
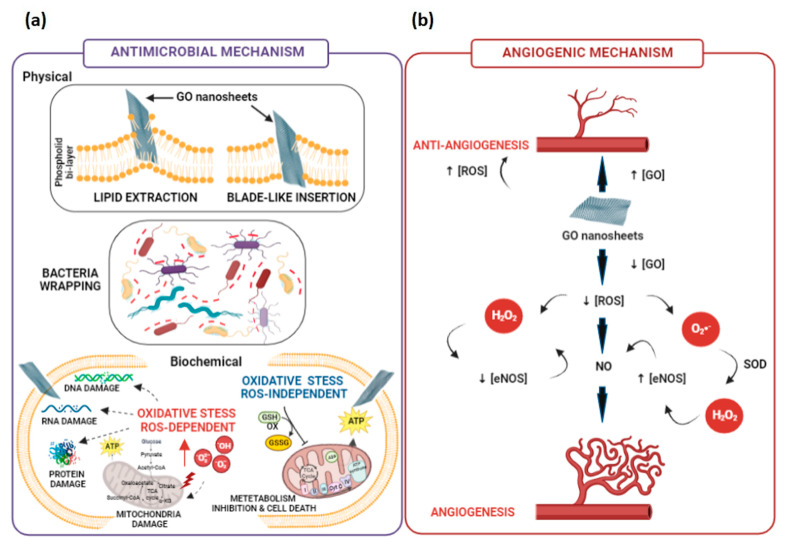
(**a**) Antimicrobial physical and biochemical mechanisms and (**b**) pro- and anti-angiogenic pathways involving GO-derivatives. Created with BioRender.com.

To date, a clear knowledge of the mechanisms underlying graphene-based nanosheets antimicrobial activity is still controversial. Several updated reviews have deeply outlined the proposed mechanisms at the cellular level, highlighting the role of the physico-chemical and structural properties of graphene as key factors influencing the interactions with selected bacteria [102,104,105]. Graphene-mediated antimicrobial activities may involve both physical and chemical mechanisms. Graphene may physically damage the bacterial membrane through direct contact with its sharp edge, causing a mechanical stress or a wrapping/trapping effect. Meanwhile, oxidative stress generated by a reactive oxygen species production (ROS-dependent) or electron transfer (ROS-independent) is the antimicrobial chemical mode of action [102,104,105] (Figure 5a). The graphene-induced bacterial cell death can be physically caused by (i) the insertion of blade-like graphene nanosheets within the phospholipid bilayer, (ii) the extraction of lipid molecules from the bilayer with consequent integrity loss, and (iii) the bacteria wrapping or trapping effect. GO interacts with bacterial cell envelopes through direct contact of its sharp edges, lining up in a parallel manner or orthogonally with respect to the membrane. GO tails are trapped within the phospholipid bilayer owing to strong van der Waals and hydrophobic bonds, leading to a spontaneous and rapid insertion of GO within bacteria envelope. GO penetration can cause a cut or deformation in the bacteria membrane, resulting in the leakage of cytoplasmic content (i.e., nucleic acids and proteins) and cell death [102,104,106,107]. This sharp edge effect mostly depends on the lateral size [108], thickness [109] and edge density [110] of GO nanosheets. Micrometer-sized graphene aligns orthogonally to the bacterial membrane, with respect to nanometer-sized graphene, which instead aligns in a parallel manner [108]. Wang et al. observed that a thicker graphene has a higher penetrating ability into bacterial lipid layer than a thinner one [109]. Conversely, Pham and co-workers proved that graphene edges’ density significantly affect the antimicrobial behaviour of the graphene nanosheets, causing the formation of pores in the bacterial cell wall, with a subsequent osmotic imbalance and eventually cell death [110]. The bacteria wrapping or trapping effect is another physical damage mechanism mediated by thin and flexible graphene nanosheets [102,104,105]. These last ones may encase bacterial cells, isolating them from the surrounding environment, preventing nutrients from passing through the cell membrane and leading to growth inhibition. Larger GO nanosheets’ bactericidal activity towards *Escherichia coli* was stronger than smaller ones, due to an easier and full covering of bacterial cells, inhibiting the proliferation [111]. Chen et al. proved that GO intertwined bacterial (*Pseudomonas syringae* and *Xanthomonas campestris*) and fungal (*Fusarium graminearum* and *Fusarium oxysporum*) pathogens have a wide range of aggregated nanosheets, resulting in a local perturbation of the phospholipid bilayer, decreasing the bacterial membrane potential and the leakage of electrolytes of fungal spores [112]. 

However, oxidative stress is deemed the most widely demonstrated chemical mechanism of graphene’s antimicrobial properties [102,104,105]. Oxidative stress interferes with bacterial metabolism by disrupting vital cellular functions and leading to cell death. It may take place through an *ROS-dependent* or an *ROS-independent* pathway. The former occurs as a consequence of an unreasonable accumulation of intracellular ROS species, such as hydroxyl radical (OH^•^), hydrogen peroxide (H_2_O_2_), singlet molecular oxygen (^1^O_2_) and superoxide anions (O_2_^•−^), which trigger protein deactivation, lipid peroxidation, mitochondrial dysfunction and bacterial cell membrane disintegration, leading to cell death. The latter arises from the charge transfer from a bacterial cell membrane to graphene disturbing, oxidizing or depleting vital cellular components and, thereby, killing the cells. Bacterial metabolism relies on several proton–electron transfer reactions, in which the simultaneous reduction of molecular O_2_ to water and adenosine triphosphate (ATP) synthesis is fundamental for bacterial cell survival. In the presence of graphene adsorbed on the surface or embedded within the phospholipid bilayer, O_2_ can be adsorbed on graphene’s edges, or its defective sites undergo reduction reactions catalysed by several reducing enzymes, interrupting the water molecule formation and producing ROS traces in the cell’s mitochondria. The expression of epoxy, hydroxyl, and carbon radical moieties on graphene surface, together with -COOH groups on the edges, strongly contributes to bactericidal effects. A graphene-dependent ROS levels increase was observed in *Xanthomonas oryzae pv Oryzae* by Chen et al. [113]; meanwhile, a different increase in ROS level depending on the nature of bacterial cell envelope was also proved: for instance, ROS levels in GO and rGO-treated *P. aeruginosa* cells were 3.8- and 2.7-fold higher than no-treated cells [114]; in addition, O_2_^•−^ levels in GO and rGO-treated *E. coli* were 2- and 1-fold higher than no-treated cells [115]. Bacterial cell membrane damage may also be mediated by lipid peroxidation, which depends on the oxidative nature of GO. Krishnamoorthy et al. observed a graphene-concentration dependence of lipid peroxidation levels enhancement in *E. coli*, *Salmonella typhimurium*, *Bacillus subtilis* and *Enterococcus faecalis* [116]. Meanwhile, GO-induced lipid peroxidation dependent on the density of defects in the carbon structure, allowing for more oxygen to be absorbed on the surface, was proved by Perreault et al. [117]. Therefore, the manipulation of the oxidative moieties on GO surface may allow for tailoring proper radical expression and the bactericidal potential. 

By exploiting the high conductivity properties of graphene, an ROS-independent oxidative stress mechanism has also been proposed. This mechanism relies on the ability of bacteria to exchange electrons from their respiratory chain on the membrane with the surrounding environment, in order to exert the bactericidal effect. Considering this, it was supposed that rGO holds a stronger oxidative capacity than GO, due to its higher conductivity [113]. However, relatively very few reports provided indirect evidence of the oxidative stress mediated by electron transfer between graphene and bacteria, revealing that graphene could oxidize glutathione or other antioxidant compounds without producing high ROS levels [106]. Later, Li and co-workers gave a more direct proof of the electron transfer mechanism by investigating the antibacterial actions of large-area monolayer graphene film on copper (Cu) conductor, Germanium (Ge) semiconductor and silicon dioxide (SiO_2_) insulator toward *S. aureus* and *E. coli* [118]. They showed that bacterial growth was significantly inhibited in the case of graphene-Cu and graphene-Ge, but not with graphene-SiO_2_. These differences could be explained assuming that an electrical circuit based on electron transfer between bacteria membrane and a conductive substrate (i.e., Cu and Ge) with the graphene takes place.

### 4.2. Angiogenic Mechanisms Involving GO-Derivatives

Angiogenesis is a complex process, also known as the process of new blood vessels formation, in which many signalling pathways are involved [119]. It includes many important steps, namely: (i) proliferation of endothelial cells by GFs, (ii) migration, and (iii) capillary tube formation. Several studies have emphasized the role of angiogenesis in different diseases, such as cancer, atherosclerosis and cardiovascular disorders. Furthermore, the process regulates embryogenesis, the menstrual cycle, and finally, the wound healing and the formation of granulation tissue, which is the topic of the present review [120]. Particularly, pro- and anti-angiogenic factors, which are naturally occurring in the body, can be used to initiate the process, following an injury. Thrombin, fibrinogen fragments, thymosin- β4, and GFs are pro-angiogenic mediators. Angiogenic GFs are sequestered inside the ECM and are stored in platelets and inflammatory cells that go through the circulation. Genes like hypoxia-inducible factors (HIF) and cyclooxygenase-2 (COX-2), which are expressed in response to inflammation and hypoxia, control the production of these substances. Indeed, wound angiogenesis is significantly influenced by hypoxia. Vascular endothelial GF (VEGF), which is found in both wound tissue and exudate, is produced as a result of the HIF-1α gene, due to the hypoxic gradient between wounded and healthy tissue. Nitric oxide (NO) generation by endothelial cells is also influenced by hypoxia. Over the past several decades, NO has emerged as a molecule of interest involved in the angiogenesis process. NO has been suggested to modulate different biological events, including vasodilation and angiogenesis, to improve local blood flow.

By contrast, angiogenesis inhibitor factors suppress blood vessel growth. While some inhibitors are stored in the ECM surrounding blood vessels, others circulate in the bloodstream at physiologically low levels. When the physiological equilibrium between angiogenesis stimulators and inhibitors is maintained, vascular growth is inhibited. However, immediately after some damage, angiogenic stimuli are delivered into the wound bed, and a variation in the vascular regulators’ levels takes place. [121]. It has been also demonstrated that wound healing response is related to a balance between ROS amount and oxidative stress species production [122]. In fact, H_2_O_2_ and O_2_ act as important redox signalling molecules in the angiogenesis process, with advantages for wound healing treatment. Furthermore, low level of ROS may activate pro-angiogenic response, whereas a high level of ROS may be anti-angiogenic [123]. In particular, ROS appears to be crucial in coordinating lymphoid cell recruitment to the wound site and efficient tissue repair because they act as secondary messengers to various immunocytes and non-lymphoid cells that are engaged in the repair process. Indeed, ROS acts in the host’s defense through phagocytes that cause a ROS burst onto the pathogens present in wounds, leading to their destruction. Excess ROS leaking into the environment during this time may have a further bacteriostatic effect [122]. Over the past several years, there is a considerable attention in understanding the GO role in the angiogenesis process. It is well known that GO is a bi-dimensional product of the oxidation and exfoliation of pristine graphene, which exhibits high biocompatibility and angiogenic properties, related to the intracellular formation of ROS. Several studies have shown that GO is able to influence immune response and angiogenic differentiation of endothelial cells, such as human vein umbilical endothelial cells (HUVECs). Moreover, GO possesses intrinsic biological properties, including antimicrobial activity, and it can control immune cell function and modulate the angiogenesis process. Particularly, it is reported that GO nanosheets have pro-angiogenic properties at low doses (1–50 ng/mL) ascribable to controlled intracellular ROS production induced by this material. Meanwhile, the material shows anti-angiogenic activity at high doses (>100 ng/mL), due to the high intracellular ROS levels generated [124]. According to Figure 5, the activity of GO may be primarily attributed to the intracellular production of ROS and reactive nitrogen species as well as the activation of phosphor-eNOS and phosphor-Akt. The understanding of these mechanisms may be important for the potential development of alternative angiogenic treatment approaches for wound care managing. In particular, GO may influence the process by modulating NO production, which plays a critical role in physiological and pathological angiogenesis. In fact, the generation of NO could be useful as pro-angiogenic therapeutic strategy, by controlling the blood pressure, the blood vessel tone and the vascular health. It is generally known that L-citrulline and NO are byproducts of NO synthesis from L-arginine by the enzyme NO synthase (NOS). Endothelial nitric oxide synthase (eNOS), among the NOS isoforms, plays a major role in endothelial cell activity. Additionally, it is widely known that H_2_O_2_ activates eNOS, which increases intracellular NO levels production [125]. H_2_O_2_ and NO thus have a direct relationship. For instance, a number of studies revealed that Akt and eNOS signalling is crucial for both angiogenesis and the proliferation of endothelial cells. Other studies have shown that eNOS is phosphorylated in an Akt-dependent manner [125]. However, even though the influence of GO (rGO) on the angiogenesis mechanism is, in part, clear, the possible interaction within the polymer matrix will be analyzed in the next paragraphs.

## 5. GO-Functionalised Cellulose-Based Nanocomposites for Wound Healing 

### 5.1. GO or rGO/Cellulose Nanocomposites

**Figure 6:**
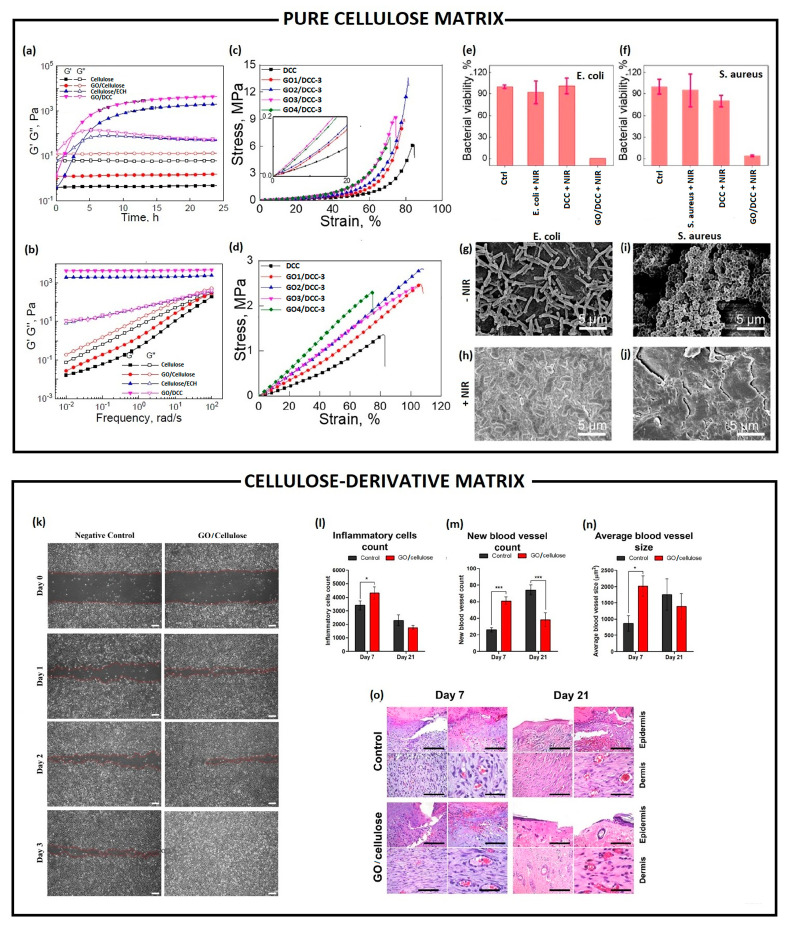
Pure cellulose matrix (top): rheological characterizations: (**a**) time- and (**b**) frequency-dependence of the storage modulus (G′) and loss modulus (G″) of a cellulose, cellulose/GO (2 wt%), cellulose/ECH (ECH:DCC molar ratio = 0.86), and cellulose/GO (2 wt%)/ECH (ECH:AGU molar ratio = 0.86). Mechanical characterizations: (**c**) compression strain–stress curves and (**d**) tensile strain–stress curves of cellulose/GO/ECH composite hydrogels with different GO contents. The insets show magnified plots of the responses in the low-strain range. Antimicrobial studies: relative percentage survival of (**e**) *E. coli* and (**f**) *S. aureus* after incubation with composite hydrogels with and without GO under NIR laser irradiation (power density of 2 W/cm^2^ under 808 nm). SEM micrographs of *E. coli* (**g**,**h**) and *S. aureu* (**i**,**j**) on DCC4-3 before (**g**,**i**) and after (**h**,**j**) NIR irradiation. Adapted from [127]. Copyright (2022), with permission from Elsevier. Cellulose-derivatives matrix (bottom): (**k**) evaluation of GO-cellulose nanocomposite effect on in vitro wound healing through scratch assay performed by using a human endothelial cell line (EA.hy926) after 0, 1, 2 and 3 days post wound induction. Red-dotted lines indicate the wound edge (scale bar = 200 µm); (**l**) inflammatory cells counts, (**m**) new blood vessels counts, and (**n**) average blood vessels size measured through ImageJ from a histological section of skin wound in rats treated with or without GO-cellulose nanocomposite for 7 and 21 days. Differences were evaluated using *one-way* ANOVA (* *p* < 0.05; *** *p* < 0.0001); (**o**) haematoxylin and eosin staining of a histological section of skin wound in rats non-treated (control) and treated with GO-cellulose for 7 and 21 days post-wound induction (scale bar = 50 µm). Reprinted from [129]. Copyright (2022), with permission from Elsevier.

Nanomaterials, like GO and its derivatives, have been successfully incorporated within cellulose-based matrixes, in the form of hydrogels or scaffolds, to simultaneously stabilise the structure of the nanocomposites and improve the bulk mechanical properties [126]. Among the works published, over the past several years, the majority of them have employed cellulose derivatives as polymer matrix; meanwhile, only one paper was focused on pure cellulose [127] (Table 4). Indeed, Wei and colleagues proposed a multi-modulus components-based strategy for preparing a double cross-linked cellulose/GO composite hydrogel (DCC), with a remarkably high strength and toughness, while retaining a high water content [127]. The authors started from pure cellulose, extracted from cotton linter pulp, dissolved in a 4.6 wt% lithium hydroxide/15 wt% urea solution. The strength and toughness of DCC hydrogel increased because of reversible non-covalent interactions (i.e., hydrogen bonding, hydrophobic and CH-π stacking) established between GO nanosheets and cellulose chains, and irreversible covalent bonds, due to the presence of epichlorohydrin (ECH). GO addition (0–8 wt%) also resulted in a higher storage and viscous moduli compared to cellulose, as reported in Table 4 and shown in Figure 6a,b). Meanwhile, ECH addition (0–3.44 mol_ECH_:mol_DCC_ ratio) allowed for speeding up the gelation time of the DCC in 2.5 h, resulting in a storage modulus several orders of magnitude higher than cellulose and DCC (Table 4). Finally, Wei et al. demonstrated that GO nanosheet content, molar ratio between GO and anhydro-glucose units of cellulose, and cross-linker agent concentration were among the critical parameters that allowed for perfectly modulating the mechanical properties of DCC hydrogels, reaching the highest stress and work of fracture in the compressive and tensile mode, as reported in Table 4 and shown in Figure 6c,d. Moreover, the authors showed that the composite hydrogels had broad-spectrum antibacterial properties versus *E. coli* (96.5%, Figure 6e) and *S. aureus* (100%, Figure 6f), upon near-infrared light (NIR) irradiation with a power density of 2 W/cm at 808 nm for 240 s, owing to GO photothermal features. Morphological studies performed via SEM revealed that the bacteria incubated with DCC hydrogels and irradiated with NIR light exhibited a fully collapsed membrane (Figure 6h,j), compared to non-irradiated *E. coli* and *S. aureus* that retained their typical rod and spherical shapes, respectively (Figure 6g,i).

**Table 4:** List of the papers focused on pure cellulose and derivatives based composites functionalised with GO(rGO) for wound healing application.

Year	Material	Cellulose Biosource	GO Processes	Crosslinker/ Gelling Agents	Inorganic/Organic Compounds Embedded	Physico-Chemical and Mechanical Properties	In Vitro Outcomes	In Vivo Outcomes	Ref.
**Pure cellulose**									
2022	GO/DCC	Cotton linter pulp	Hummers method	ECH:DCC	-	σ_comp_ = 0.05–13.6 Mpa; w_comp_ = 0.05–1.47 MJ/m^3^); σ_tens_ = 0.02–2.8 Mpa; w_tens_ = 0.01–1.49 MJ/m^3^ G′ = 0.4–1.5 Pa (GO effect) G″ = 5.8–12.8 Pa (GO effect) G′ = 2037 Pa at 1 rad/s (ECH effect)	*E. coli* death 96.6% *S. aureus* death 100% (8% wt GO, NIR irradiation 2 W/cm, 808 nm, 240 s)	-	[127]
**Cellulose-derivatives without bioactive compounds**									
2019	rGO/CMC	chemically modified	rGO oxidation by NaOH				HDF cells viability (>90%) *S. aureus* biofilm reduction 81–84%. *P. aeruginosa* biofilm reduction 51–62% *S. aureus* biofilm thickness reduction 47%, *P. aeruginosa* biofilm thickness reduction 40%	Higher biofilm inhibition in *C. elegants* infected with *S. aureus* than *P. aeruginosa*.	[128]
2021	GO/CMC	chemically modified	Hummers method				EA.hy926 migration promotion and increasing of the wound closure rate	Increase of newly formed blood vessels in number and in size. Highly densely packed collagen deposition.	[129]
**Cellulose-derivatives with bioactive compounds**									
2019	AGO/HPC	chemically modified	Hummers method		Ag/ZnO	σ_tens_ = 19.4–28.8 Mpa	*E. coli* inhibition zone 11.25–12.32 mm; *S. aureus* inhibition zone 12.18–13.76 mm	Reduction of wound size after 8 days. Newborn blood vessels filled with red blood cells after 12 days	[130]
2021	GO/TiO_2_/Cur/CA	chemically modified	GO/TiO_2_ nanocomposite by hydrothermal method		TiO_2_ Cur	σ_tens_ = 35.45 Mpa; swelling ≈ 50–55%	*E. coli* inhibition zone 19 ± 0.2 mm; *S. aureus* inhibition zone 17 ± 0.1 mm; *P. aeruginosa* inhibition zone 16.4 ± 0.2; *E. faecalis* inhibition zone 14 ± 0.2 mm. Wound closure 77 ± 4.18% (12 h)–96 ± 3.26% (24 h)		[131]
2022	O/NA/CA	chemically modified	GO pyrolyzation of cellulose		NA (98%)	σ = 2.46 ± 0.022–4.94 ± 0.027 (·10^−5^ Mpa); ε = 53.95 ± 0.52–19.05 ± 0.24 %; E = 4.56–25.93 (10^−7^ Mpa).	HeLa cells viability (>80%) *S. aureus* inhibitory zone 9.15 mm		[132]
2022	GO/Cys/DAC GO/Meth/DAC GO/Cys/Meth/DAC	chemically modified			Cys and Meth		*B. subtilis* inhibition zones (23 ± 0.53 mm and 17 ± 1.27 mm), *S. aureus* inhibition zones (21 ± 0.58 mm and 11 ± 0.69 mm), *E. coli* inhibition zones (12 ± 0.51 mm and 19 ± 1.01 mm), *P. aeruginosa* inhibition zones (24 ± 0.50 mm and 27 ± 0.95 mm), *C. albicans* inhibition zones (12 ± 0.53 mm and 23 ± 0.87 mm) and *C. neoformans* inhibition zones (22 ± 0.52 mm and 32 ± 0.93 mm) for DAC/GO/Cys and DAC/GO/Meth, respectively.		[133]
**Cellulose with polymeric blend**									
2018	rGO/PVA/CMC	chemically modified	rGO reduction under sunlight via convex lens	PVA			EA.hy926 proliferation improved up to 72 h	Number of blood vessels increase 25–62% Blood vessels thickness increase 18.4–44.5%	[134]
2020	rGO/Ag/PU/Cur/CA	chemically modified	Hummers method	PU	rGO/Ag Cur	σ_yield_ = 0.7 Mpa, σ_max_ = 3.75 Mpa, and E = 0.41 (rGO effect) σ_yield_ = 0.4 Mpa, σ_max_ = 3.4 Mpa, and E = 0.29 (curcumin effect)	*P. aeruginosa* death 100%, and *S. aureus* death 95%.	Wound healing ratio 100%. Collagen fibers deposition after 15 days	[135]
2020	Ag-ZnO@GO/k-car/KG/CMC	chemically modified	Hummers method	k-car	Ag-ZnO KG	σ_comp_ ≈ 200 kPa; E ≈ 150 Mpa	L929 viability 100 % after 7 days. *S. aureus* death 96% and *E. coli* death 98%.	Faster ri-epithelialization process	[136]

**Abbreviations**: GO, graphene oxide; DCC, double crosslinked cellulose; ECH, epichlorohydine; σ_comp_, stress at fracture; w_comp_ fracture strain; σ_tens_, tensile strength; w_tens_ work of fracture; G′, storage modulus; G″, viscous modulus; NIR, near-infrared radiation; MC, methylcellulose; EA.hy926, human vascular endothelial-derived cell line; rGO, reduced graphene oxide; CMC, carboxymethyl cellulose; HDF, human dermal fibroblasts; CA, cellulose acetate; NA, naproxen; σ, stress; ε, strain; E, Young’s modulus; HeLa, human tumoral epithelial cell; DAC, dialdehyde cellulose; Cys, cysteine; Meth, methionine; TiO_2_, titanium dioxide; Cur, curcumin; HPC, hydroxypropyl cellulose; AGO, GO-grafted silver coated zinc oxide; Ag, silver; ZnO, zinc oxide; PVA, polyvinyl alcohol; PU, polyurethane; σ_yield_, ultimate strength; k-car, k-carrageenan; KG, Konjac Glucomannan; L929, murine dermal fibroblasts.

Differently from Wei et al., Ali et al. and Soliman et al. developed a GO/cellulose nanocomposite, by using water-soluble MCC [128,129]. Ali and co-workers employed rGO (50, 100, 250, 500 µg/mL), as filler into a water-soluble sodium CMC (NaCMC) hydrogel formulation as functional antibiofilm wound dressing [128]. The rGO/CMC hydrogels exhibited a synergistic antibiofilm effect, inhibiting biofilm formation in *S. aureus* (81–84%) and *P. aeruginosa* (51–62%), respectively. By contrast, the authors reported that GO/CMC hydrogels induced a significant increase in the biofilm formation by *S. aureus* (270%) and *P. aeruginosa* (63%), due to the high level of oxygenated functional groups, which might favour bacteria adhesion. rGO/CMC hydrogel could act in preventing bacteria adhesion in the early adsorption stage because they could be trapped and stacked within rGO agglomerated structures dispersed into the CMC hydrogel matrix. Additional qualitative analysis by using confocal laser scanning microscopy showed that the biofilm thickness was reduced by up to 47% in *S. aureus* and 40% in *P. aeruginosa*, appearing as disintegrated and poorly defined microcolonies. Similarly, GO nanosheets were intercalated within cellulose chains, in a weight ratio 1:1, through non-covalent bonds (i.e., van der Waals, electrostatic and hydrogen bonds) by Soliman and co-workers [129]. GO/MCC nanocomposite retained the viability of human EA.hy926 endothelial cells over time, and enhanced their migration capability in wound scratch assay within 1–3 days (Figure 6k). In addition, GO/MCC significantly promoted the wound healing process in the in vivo rat skin wounds, over the treatment time. Within a short time (day 7), a significant influx of inflammatory cells (Figure 6l) was observed in the dermal region, with a significantly higher number of newly formed vessels (Figure 6m) followed by a significant increase in the size of the blood vessels (Figure 6n) in wounds. Meanwhile, within a longer time (day 21), a mature/remodelled dermis presenting skin appendages (such as hair follicles and sebaceous glands) was observed, with a higher amount of densely arranged collagen fibres (Figure 6o). The authors hypothesized that GO/MCC nanocomposites triggered the in vivo repairing of wounds through GO’s capability of scavenging ROS in the wound area, regulating the inflammation and thus driving the healing.

**Figure 7:**
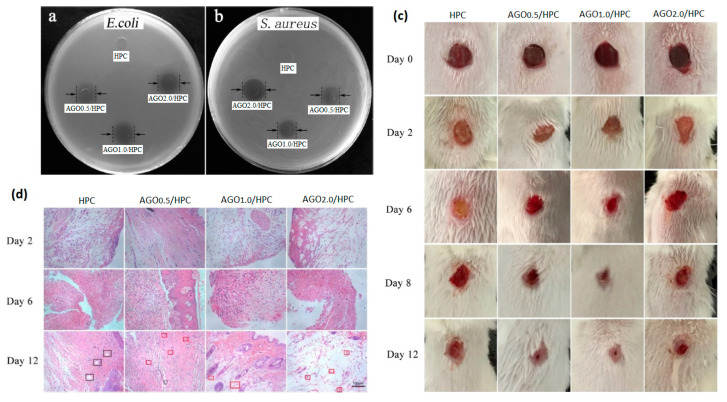
Inhibition zone on plates with solid-state nutrient agar against (**a**) *E. coli* and (**b**) *S. aureus* showing the corresponding diameters of the inhibition zone induced by HPC, AGO_0.5_/HPC, AGO_1.0_/HPC and AGO_2.0_/HPC; (**c**) in vivo BALB/C mouse wound healing model with a 6 mm-diameter of wound on the back, infected with *S. aureus* (1.0·10^7^ CFU/mL) and treated with HPC, AGO_0.5_/HPC, AGO_1.0_/HPC and AGO_2.0_/HPC for 0, 2, 6, 8 and 12 days; (**d**) haematoxylin and eosin staining of histological section of skin wound in mice treated with HPC, AGO_0.5_/HPC, AGO_1.0_/HPC and AGO_2.0_/HPC for 2, 6 and 12 days post-wound induction (scale bar = 100 µm). Adapted from [130]. Copyright © 2019 American Chemical Society.

With the aim of improving the inherent potential of the GO and rGO/cellulose nanocomposite as delivery systems, several attempts were investigated by incorporating bioactive compounds (i.e., drugs, amino acids, extracts, nanoparticles, etc.). In this regard, Wang et al. proposed multifunctional composite films consisting of hydroxypropyl cellulose (HPC) matrix and GO-grafted silver-coated zinc oxide nanofillers (AGO) by solution blending [130]. GO was covalently grafted onto the surface with Ag/ZnO by using isophorone diisocyanate, as a coupling agent, which involved the -OH and the -COOH groups on the GO surface and the -OH groups on the Ag/ZnO surface. The AGO intercalation within the HPC matrix contributed to enhancing inter-molecular interactions, resulting in an AGO concentration-dependence increase of the tensile strength (see Table 4). In addition, AGO/HPC films significantly inhibited *E. coli* and *S. aureus* growth with a AGO concentration-dependence (Figure 7a,b). The authors showed that the antimicrobial activity observed in the AGO/HPC films was due to: (i) the generation of OH• and HO_2_• radicals, by ZnO nanoparticles upon high energy irradiation, which could destroy the microbial cells’ structure [137], followed by (ii) Zn ions penetration through the bacteria cells wall and the reaction with the intracellular active protease [138,139]; (iii) Ag^+^ ions interaction with bacteria cell wall, altering its permeability and entering into the cytoplasm promoting the oxidative stress and killing the cells [140]. An in vivo BALB/C mice skinny wound model confirmed a synergistic antibacterial effect and accelerated wound-healing of the AGO/HPC films after 12 days (Figure 7c). Histological images showed many white blood cells and neutrophils recruited in a wound site when bacterial infection occurred in vivo. Over time, new blood vessels filled with red blood cells appeared as highlighted by a red rectangle in Figure 7d. A reliable alternative approach by integrating curcumin (Cur) and GO-grafted titanium dioxide (TiO_2_) nanocomposite into CA nanofibers was proposed by Prakash and colleagues. The authors processed the biomaterial by electrospinning, for eradicating the drug-resistant bacteria [131]. GO/TiO_2_ nanocomposite incorporation decreased the viscosity of the CA solution, while increasing the conductivity, hence resulting in nanofibers’ diameter decrease (≈180 nm). As expected, the integrated GO/TiO_2_/Cur/CA nanofibers showed a lower swelling capacity (50–55%) than CA (75–85%) after 7 days, due to strong hydrogen bonding between Cur, GO and CA. However, GO and GO/TiO_2_ nanocomposites incorporation in CA matrix created polymeric interactions that hindered the incision sites for triggering the degradation process. Meanwhile, the Cur addition allowed for increasing the degradation. Furthermore, GO/TiO_2_/VA nanofibers exhibited a higher tensile strength compared to GO/CA, due to -COOH and -OH functional groups which resulted in intermolecular hydrogel bonding with the CA matrix, whereas Cur addition worked as a nanofiller (discontinuous phase) within a continuous matrix (nanofiber), increasing the density and the mechanical strength. Finally, the GO/TiO_2_/CA nanofibers proved the simultaneous antibacterial activity against wounds pathogens and potential wound healing capability. GO/TiO_2_/Cur/CA nanofibers showed the highest zone of inhibition. The antimicrobial activity took place through the combined capability of TiO_2_ and GO of increasing the ROS-mediated oxidative stress and leading to cell death [141] on one hand; and the possible interference of Cur with the FtsZ protein functions, involved in bacterial cytokinesis, on the other hand, resulting in the bacterial growth inhibition [142,143]. Finally, the GO/TiO_2_/CA nanofibers not only proved to be biocompatible, but above all they demonstrated to promote the NIH3T3 fibroblasts proliferation and migration, helping in the closure of wounds of 77% already at 12 h. On the other hand, Purnamasari et al. investigated the potential improvements achieved by simultaneous incorporation of GO and a commercial drug, such as Naproxen (NA), in CA-based hydrogel formulation processed in the form of nanofibers by electrospinning [132]. GO fillers enhanced the mechanical strength of the fibres, driving the production of fibres with a lower diameter (≈447 nm), higher conductivity (1.5 µS/cm) and keeping the NA onto the nanofiber. As the GO increased (0, 0.1, 0.5, 1 g), a corresponding increment in the stress and a reduction in the fibre’s strain ability were recorded, thus resulting in Young’s modulus increase (Table 4). Even though GO was strictly embedded into the nanocomposite matrix due to a purely physical interaction, it showed the ability to strengthen the nanofibers. GO/CA composite nanofibers were able to sustain the viability of HeLa cells above 80% over 7 h. By contrast, the addition of NA increased HeLa cells’ death already after 1 h with a CC_50_ (concentration that reduced the cells proliferation by 50%) of 29.33 µg/mL. However, it showed a strong antibacterial activity against *S. aureus*, with an inhibition zone of 9.15 mm. Concurrently, Hashem et al. developed a potential antimicrobial, antiviral and biocompatible nanocomposite based on DAC, GO and sulphur-containing aminoacids, such as cysteine (Cys) and methionine (Meth) [133]. Both GO/Cys/DAC and GO/Meth/DAC proved a higher and promising antimicrobial activity against Gram^+^ (*B. subtilis*, *S. aureus*), Gram^−^ (*E. coli*, *P. aeruginosa*) and unicellular fungi (*Candida albicans*, *Cryptococcus neoformans*). The antimicrobial activity of GO/DAC nanocomposite was improved by the presence and availability of protonated aminoacid residues, which could interact with the bacteria membrane and induce the death. 

**Figure 8:**
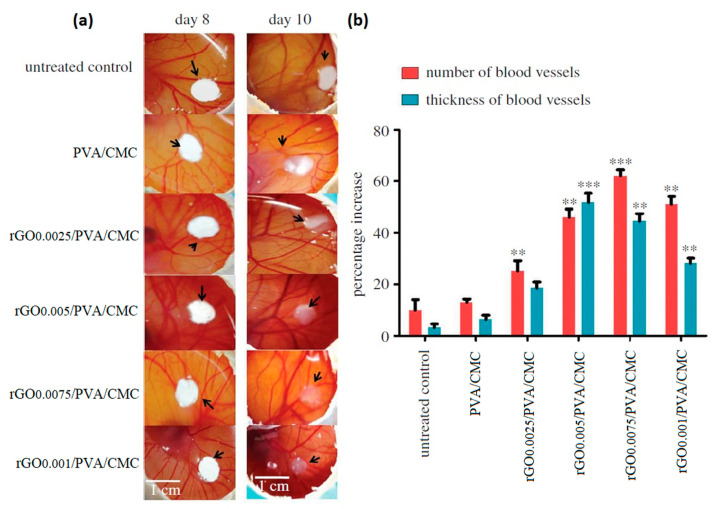
(**a**) Digital photos of chick chorioallantoic membrane (CAM) model untreated and treated with PVA/CMC and PVA/CMC with 0.0025, 0.005, 0.0075 and 0.01% *w*/*v* rGO; (**b**) percentage increase in the number (red bars) and (green bars) blood vessels after 10 days of CAM model (the value are normalised to untreated control at 8 days, ** *p* < 0.01, *** *p* < 0.001 vs. control). Adapted from [134]. Copyright © 2018 by the Royal Society under the terms of the Creative Commons Attribution License.

Another possible approach to improve the mechanical strength and toughness of GO/cellulose nanocomposites may involve the use of synthetic or natural polymers as blends [134,135,136]. Chakraborty et al. synthesised a porous scaffold by freeze-drying, starting from a polymeric blend of polyvinyl alcohol (PVA) and CMC with different amounts of rGO (0, 0.0025, 0.0005, 0.0075 and 0.01% *w*/*v*) [134]. The role of rGO was explored in terms of angiogenesis enhancement. The authors observed that rGO interacted via non-covalent bonding with the polymeric blend, without altering any physico-chemical property of PVA/CMC matrix. NIH3T3 and EA.hy926 cells’ good adhesion and proliferation confirmed the biocompatibility of rGO/PVA/CMC scaffolds. Notably, rGO/PVA/CMC with 0.0005 and 0.0075% of rGO successfully increased the EA.hy926 cells proliferation at 72 h, probably due to the slow release of rGO from the scaffold into the culture medium. The pro-angiogenic features of rGO/PVA/CMC were validated in vivo by using the chick chorioallantoic membrane (CAM) model. Two days after scaffolds implanting on the CAM of a developing chick embryo, angiogenesis was remarkably increased (Figure 8a): rGO/PVA/CMC with 0.0005, 0.0075 and 0.01 % rGO induced a significant increase in the number of blood vessels (Figure 8b), along with an absolute increase in blood vessel thickness up to 51.7 % in correspondence with rGO/PVA/CMC with 0.005% rGO. This latter is an evident sign of the newborn blood vessels’ maturation, due to a phenomenon called arteriogenesis [144]. The authors ascribed this pro-angiogenic effect to the rGO ability of increasing the intracellular ROS levels [145], triggering the biochemical pathways involved both in the angiogenesis and in the arteriogenesis [145].

Later, Esmaeili et al. and Li et al. adopted the same approach by a co-encapsulation of GO/metal oxide nanoparticles, along with a bioactive agent, within a polymeric blend of cellulose derivatives for improving both the mechanical properties and wound healing performances [135,136]. Particularly, Esameili et al. designed a polyurethane (PU)/CA electrospun nanofiber mat, embedding Cur and rGO/Ag [135]. The co-embedding of rGO/Ag and Cur in the PU/CA nanofiber matrix reduced the fibre diameter up to 222 ± 44 nm. Meanwhile, rGO/Ag and Cur showed an opposite effect on the mechanical properties: rGO/Ag enhanced the tensile strength, acting as a nanofiller, while Cur decreased it (Table 4). In addition, the nanocomposite mat with and without Cur exhibited significant antibacterial activity, leading to an inactivation rate of 100% in *P. aeruginosa* and 95% in *S. aureus*, owing to the simultaneous release of Ag ions, which may destroy the bacteria membrane, and the Cur, which can inhibit the shikimate pathway for aromatic amino acid synthesis [142] or the pathway for the protofilament developing [143]. Finally, in vivo histopathological imaging of rat wound skin proved that nanofiber mats significantly stimulated the wound healing process: high level of adnexal growth, mature collagen fibres precipitation and neovascularization were observed after 15 days. Similarly, Li et al. prepared an Ag-ZnO loaded CMC/k-carrageenen/GO/konjac glucomannan hydrogel to validate its efficacy in the antimicrobial and regenerative processes involved in the wound healing [136]. The hydrogel showed a great swelling capability (≈200%), even if the Ag-ZnO nanoparticles addition reduced the swelling compared to the matrix without nanofiller, but mostly high mechanical properties due to a uniform distribution of the Ag-ZnO nanoparticles within the matrix establishing a solid interfacial connection mediated by strong hydrogen bonds. The hydrogel exhibited a strong antibacterial activity, by reducing the viability of *S. aureus* by 96% and *E. coli* by 98%, while it sustained the viability of L929 murine fibroblasts, stimulating their proliferation up to 7 days. Finally, the hydrogel demonstrated promising efficacy in healing an in vivo diabetic ulcer mouse model, promoting a more rapid re-epithelialization process as early as 8 days.

### 5.2. GO or rGO/Bacterial Cellulose Nanocomposites

Over the past years, BC has been also employed as polymer matrix in combination with GO (rGO) for wound healing applications and the last 3-years advances are summarized in Table 5. A mussel mimetic transdermal patch based on BC was prepared by Khamrai et al. [12]. In particular, BC was modified via an amidation reaction between the carboxylated BC and dopamine (DOPA), a catechol-containing compound. Furthermore, the free -OH group of the DOPA moiety was used to prepare rGO/Ag-NPs/DOPA/BC. rGO is able to confer excellent thermal, electrical and mechanical features. In particular, an increase in the tensile strength was achieved by incorporating 0.2 g of rGO and 2 g of DOPA/BC into 20 mL solution. The antimicrobial action of the prepared film was determined against both Gram^+^ (*S. aureus* and *Lysinibacillus fusiformis*) and Gram^−^ (*E. coli* and *P. aeruginosa*) bacteria. Results showed that rGO/Ag-NPs/DOPA/BC exerted a clear bactericidal activity, mainly due to the presence of Ag-NPs. The authors found that the cell surface of *E. coli* and *S. aureus* was drastically altered, and the cell wall was squeezed in the presence of rGO/Ag-NPs/DOPA/BC, indicating its significant antimicrobial activity. The cell compatibility of the composite film over the NIH 3T3 fibroblast cell line was assessed through an MTT assay. The in vitro wound-healing assays over the NIH 3T3 cell line and A549 human lung epithelial cell line revealed that the presence of rGO and Ag-NPs in the composite film accelerated the wound healing process [12]. GO-copper oxide (CuO)/BC nanocomposites were prepared by Xie et al. [146] by mixing GO-CuO solution at different concentrations with BC slurries (1% dry weight). Particularly, GO-CuO (2.5, 5 and 7.5 mg/mL) was introduced to endow antibacterial properties. The proportion of GO-CuO in GO-CuO/BC composites ranged from 5% to 15% (*w*/*w*%). In this case, the presence of CO provided nucleation sites for in situ CuO nanosheets growth. Results from microbiological tests showed that the CuO/BC nanocomposites displayed better antimicrobial properties with higher activity against Gram^+^ than Gram^−^ bacteria, underlying the synergic contribution of GO and CuO into the polymer matrix [146]. The authors also tried to elucidate the mechanisms of antimicrobial activity of the nanocomposites, which could be due to (i) a direct contact and interaction of GO sharp nanosheets and CuO nanorods with the cell membrane and lipid membrane, resulting in alteration of membrane permeability, (ii) the disruption of the integrity of the bacterial membrane, accompanied by cellular deformation, surface collapse, surface perforation and roughness, and (iii) an increased production of ROS leading to cell death. Moreover, the GO/CuO nanohybrid-decorated cellulose nanocomposite showed good cell compatibility in vitro towards mice fibroblast cells [146]. Zhang et al. prepared a GO/BC composite film modified with DOPA and immersed in a silver solution [147]. The material was conductive and produced a weak current, generating heat when a voltage was applied. This allowed it to accelerate wound cell migration and promote wound healing. In addition, Ag-NPs immobilized on the surface released Ag^+^, which generated many oxidizing free radicals that killed bacteria. The in vitro cytotoxicity tests showed that the composite film had excellent biocompatibility, giving it good application prospects for wound dressings [147].

**Figure 9:**
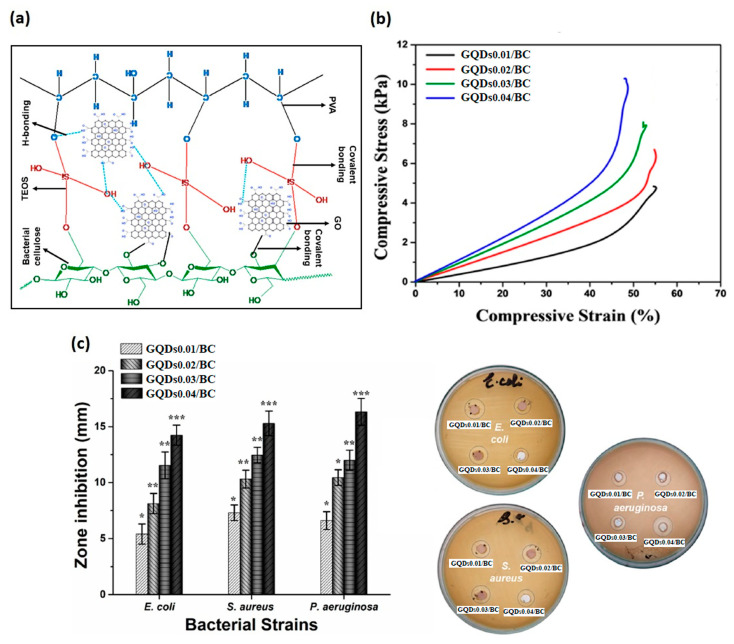
(**a**) The proposed chemical interaction of the bacterial cellulose, polyvinyl alcohol, GO, and crosslinked via TEO; (**b**) stress–strain curve of hydrogels; (**c**) the antibacterial activities of composite hydrogels against different severe skin infections causing Gram^+^ and Gram^−^ pathogens. * *p* < 0.05, ** *p* < 0.01 and *** *p* < 0.00. (GQDs_0.01_/BC, GQDs_0.02_/BC, GQDs_0.03_/BC, and GQDs_0.04_/BC) were assigned to these composite hydrogels after a different GO amount (0.01, 0.02, 0.03, and 0.04 mg). Adapted from [3]. Copyright © 2022 by the authors. Licensee MDPI, Basel, Switzerland.

For possible application in the treatment of wounds, BC was impregnated with GO quantum dots (GQDs) by Zmejkoski et al. [148]. Successful loading of approximately 11.7 weight percent of GQDs into the BC was achieved. The GQDs/BC composites demonstrated excellent water and wound fluid absorption, which is in line with good dressing qualities. The biomaterials significantly inhibited *S. aureus* and *S. agalactiae* and had bactericidal effects on *E. coli* and *P. aeruginosa* that were resistant to methicillin. The capacity of the GQDs to interact with bacterial membrane or cell wall, which results in destabilization of its integrity, is associated with the bactericidal effect [148]. The in vitro healing research revealed a significant migration of human fibroblasts following the application of GQDs/BC hydrogels. Furthermore, eNOS, VEGF A, matrix metallopeptidase 9, and vimentin gene expression in fibroblasts were noticeably elevated after 72 h of exposure to GQDs/BC, supporting angiogenesis [148]. Finally, Al-Arjan et al. reported a systematic study by using GO functionalized BC, which was obtained by hydrothermal treatment [3] (Figure 9a). Different concentrations of GO (0.01, 0.02, 0.03 and 0.04 mg) were employed. The presence of GO increased the surface roughness. This aspect is of paramount importance in wound healing, as the surface roughness helps wounds by providing them with important hydration. However, there was a threshold limit behind which a GO amount can cause cracking on drying. Furthermore, wettability as well as the swelling increased by increasing GO concentrations. It is worth noting that the increase of GO concentration allowed for improving mechanical properties (Figure 9b) and decreasing the biodegradation as the GO acted as a crosslinker and as a reinforcement of the polymer matrix [3]. The antibacterial capabilities were tested against microorganisms that cause Gram^+^ and Gram^−^ severe infections. By increasing GO concentration, a maximum antibacterial effect was observed (Figure 9c). It can be because the sharp edges of the maximal GO quantity tear the bacterial membrane. In order to prevent bacterial development, the polymeric component of the hydrogel may interact with the bacterial membrane by surrounding and penetrating a number of accessible functional groups [3]. By increasing the quantity of GO, these composite hydrogels demonstrated promising anticancer effects against the U87 cell line. In addition, the biggest anti-cancer effect was observed in the Cur loaded-BC that had the highest GO concentration. This might be as a result of the composite hydrogel’s interaction with the cellular membrane, which enabled Cur to carry out anticancer actions. The research findings allowed for concluding that these composite hydrogels have controlled medication release, physico-mechanical, and intrinsic antimicrobial qualities, which make them an appropriate biomaterial for skin wound healing [3]. 

## 6. Conclusions and Future Perspectives

In the last several decades, several research groups have focused on the development of multifunctional GO or rGO/cellulose nanocomposites, in the form of injectable hydrogels or scaffolds for wound healing applications. In particular, several optimized dressings have been specifically designed exploiting the interesting structural and physico-chemical properties of cellulose (i.e., water retention, flexibility, toughness), and the potential antimicrobial and angiogenic effect of GO and rGO. However, despite the encouraging results achieved, there are still several questions that have to be answered and different limitations to overcome. In particular, even though the role of GO or rGO in triggering the bactericidal and pro-/anti-angiogenic properties is well-known when used alone, further investigations are required to understand its role when it is embedded within 3D cellulose-derived matrixes. Indeed, among the studies herein reported, only a few have tried to hypothesise the mechanism of action and also validate their efficacy through suitable in vivo models. However, deeper studies of the main molecular pathways, along with biodegradation and bioaccumulation of the organic and inorganic components in vivo, could be helpful. Furthermore, the presented literature lacks nanocomposites fully based on GO or rGO and cellulose obtained from agricultural or industrial wastes. Indeed, considering the increasing interest in the use of sustainable raw materials, the creation of such nanocomposite biomaterials might represent a promising starting point in the development of innovative smart dressings for wound healing applications.

## Figures and Tables

**Table 1:** Cellulose content within different biomasses from agricultural waste.

Agriculture	Cellulose (%)
Wood	35–50
Wheat straw	33–40
Switchgrass	30–50
Bagasse	44
Olive husk	24
Sunflowers	26
Rice straw	33
Rice husk	49
Cotton	80–95
Nutshells	25–30
Banana fibers	60–65
Corn cob	42–45
Oat straw	33–35
Hazelnut shell	29

**Table 2:** Common cellulose derivatives.

Derivative	R_1_	R_2_	R_3_
Carboxymethyl cellulose (CMC)	COONa	COONa	COONa
Methylcellulose (MC)	CH_3_	CH_3_	CH_3_
Dialdehyde cellulose (DAC)	H	C=O	C=O
Hydroxyethyl cellulose (HEC)	CH_2_CH_2_OH	CH_2_CH_2_OH	CH_2_CH_2_OH

**Table 3:** List of GO preparation approaches from agricultural and industrial wastes.

Year	Waste	Physico-Chemical Process and Reagents	References
**Agricultural Wastes**			
2015	Sugarcane bagasse	Muffle furnace (T = 300 °C, t = 10 min) Ferrocene	[91]
2020	Coconut shell	Carbonization (T = 600 °C, t = 3 h) + Modified Hummers method (NaNO_3_, H_2_SO_4_, KMnO_4_)	[93]
2020	Tea	Carbonization (T = 750 °C, t = 3 h, argon) + Modified Hummers method (NaNO_3_, H_2_SO_4_, KMnO_4_)	[92]
**Industrial Wastes**			
2018	Carbon Tyre	Crushing + Modified Hummers method (NaNO_3_, H_2_SO_4_, KMnO_4_)	[94]
2019	Toner Powder	Modified Hummers method (NaNO_3_, H_2_SO_4_, KMnO_4_)	[95]
2020	Generic	Leaching (6 M HCl, T = 70 °C, t = 210 min) + Modified Hummers method (NaNO_3_, H_2_SO_4_, KMnO_4_)	[96]

**Table 5:** List of the papers focused on bacterial cellulose based composites functionalised with GO(rGO) for wound healing application.

Year	Material	Cellulose Biosource	GO Processes	Crosslinker/ Gelling Agents	Inorganic/Organic Compounds Embedded	Physico- Chemical and Mechanical Properties	In Vitro Outcomes	In Vivo Outcomes	Ref.
2019	rGO/Ag/DOPA/BC	*Gluconacetobacter xylus* (MTCC7795)	Hummers method	--	AgNO_3_	σ_tens_ = 1–5.52 ± 0.07 MPa σ_tens_ = 5.21 ± 0.03 MPa (Ag NPs effect) Average resistance of 84 kΩ	*E. coli* inhibition zone diameter 15 ± 1 mm; *P. aeruginosa* inhibition zone diameter 11 ± 0.5 mm, *S. aureus* inhibition zone diameter 13 ± 0.5 mm, *L. fusiformis* inhibition zone diameter 14 ± 0.75 mm. High stimulation of NIH3T3 proliferation favouring the wound closure (18 h).	-	[12]
2019	GO-CuO/BC	*Gluconacetobacter xylinus* (traditional Chinese drink)			CuO		*S. aureus* inhibition zone diameter 16.3–18.3 mm; *E. coli* inhibition zone diameter 12.7–15.2 mm, *B. subtilis* inhibition zone diameter 27.8–28.5 mm, *P. aeruginosa* inhibition zone diameter 0–15.2 mm. NIH3T3 viability > 100%	-	[146]
2021	rGO/Ag-pDA/BC	*Gluconacetobacter xylinus*	Hummers method		AgNO_3_		*E. coli* inhibition zone 6.3 mm (AgNO_3_ effect)	-	[147]
2022	GQDs/BC	*Komagataeibacter oboediens* (IMBG180)			-		Bacterial inhibition against *S. aureus*, *MRSA*, *E. coli*, *P. aeruginosa* and *S. agalactiae*. Bactericidal effect against *MRSA*, *E. coli* and *P. aeruginosa.* Angiogenesis stimulation validated by up-regulation of eNOS, VEGFA, MMP-9 and vimentin		[148]
2022	GO/PVA/BC	-		TEOS PVA	Cur	Increase of the compressive stress and hydrophilicity by increasing the GO content	*E. coli* inhibition zone 5–15 mm; *S. aureus* inhibition zone 7.5–16 mm; *P. aeruginosa* inhibition zone 7–17 mm		[3]

**Abbreviations**: BC, bacterial cellulose; DOPA, dopamine; AgNO_3_, silver nitrate; NIH3T3, mouse embryonic fibroblast cell line; CuO, copper oxide; pDA, polydopamine; GQDs, graphene oxide quantum dots; MRSA, Methicillin-Resistant Staphylococcus Aureus; TEOS, Tetraethyl orthosilicate.

## Data Availability

Not applicable.
